# The complete chloroplast genome of *Erodium cicutarium* (Linnaeus) l’ Héritier ex Aiton 1789 (Geraniaceae): genome characterization and phylogenetic consideration

**DOI:** 10.1080/23802359.2024.2420847

**Published:** 2024-10-24

**Authors:** Jiawen Cui, Shengwei Chen, Yujie Wu, Ting Guo, Lei Zhang

**Affiliations:** Key Laboratory of Ecological Protection of Agro-pastoral Ecotones in the Yellow River Basin National Ethnic Affairs Commission of the People’s Republic of China, School of Biological Science & Engineering, North Minzu University, Yinchuan, Ningxia, P. R. China

**Keywords:** *Erodium cicutarium*, Geraniaceae, chloroplast genome, inverted repeat regions loss, phylogenetic analysis

## Abstract

*Erodium cicutarium* is an annual herbaceous plant valued for its applications in traditional medicine. However, the chloroplast genome of *E. cicutarium* has yet to be reported. In this study, we assembled chloroplast genomes of *Erodium cicutarium* using Illumina sequencing reads. The chloroplast genome was 114,652 bp long, harbored 111 complete genes, and its overall GC content was 39.1%. In Maximum Likelihood (ML) and Bayesian inference (BI) trees, the 13 *Erodium* species divided into three main clades, with *E. cicutarium* and *E. carvifolium* forming a monophyletic group, suggesting a close relationship between the two species. The *E. cicutarium* cp genome presented in this study lays a good foundation for the *Erodium*.

## 1. Introduction

*Erodium cicutarium* (Linnaeus) L’ Héritier ex Aiton 1789 is an annual herbaceous plant that belongs to the genus *Erodium* (Geraniaceae). It primarily thrives in meadows, floodplains, and gravel areas (700–2200 m a.s.l.) across various regions of China (Wu et al. 2008; Kubitzki 2014). Additionally, as a traditional Chinese medicine, various components of *E. cicutarium* have been used for their medicinal properties, including astringent, hemostatic, diaphoretic, diuretic, anti-inflammatory, topical disinfectant and lactation-promoting effects (Lis-Balchin and Hart 1994; Radulović et al. 2009; Ali and Al 2017). According to literature, phenolic compounds are the primary bioactive compounds in *E. cicutarium* (Munekata et al. 2019). Tannins, flavonoids, and organic acids are the most frequently isolated compounds from this species (Sroka et al. 1994; Penkov et al. 2014).

The chloroplasts are important plant organelles that possess a small genome (cp genome) playing a vital role in photosynthesis, biosynthesis, and carbon sequestration (Li et al. 2015; Hollingsworth et al. 2016). Compared to the nuclear genome, the chloroplast genome (CPG) is smaller in size, has a low nucleotide substitution rate, exhibits single-parental inheritance, and is haploid nature. Since the first chloroplast genome was sequenced from *Nicotiana tabacum* (Sugiura et al. 1986), the complete chloroplast genome has increasingly addressed phylogenetic issues (Guo et al. 2017; Zhang et al. 2018; Xie et al. 2022; Ran et al. 2024). Besides evolutionary aspect, chloroplast genomes have been extensively utilized to resolve lingering queries in plant taxonomy (Hu et al. 2016; Yang et al. 2022). A comprehensive examination of *E. cicutarium* could have significant implications for understanding the origin and evolution of the Geraniaceae. Previous phylogenetic investigations of *Erodium* primarily relied on a limited number of chloroplast genes or ITS (Bakker et al. 2006; Fiz et al. 2006; Herrmann and Wink 2014), and investigations into the genomic characteristics of *Erodium* remain underexplored, and the chloroplast genome of *E. cicutarium* has yet to be reported.

In this study, we assembled and analyzed the complete chloroplast genome of *E. cicutarium* for the first time. Our objectives were (1) to characterize the structural features of the chloroplast genomes of *E. cicutarium*, and (2) to elucidate the evolutionary relationships of *E. cicutarium*

## 2. Materials

**Figure 1.**
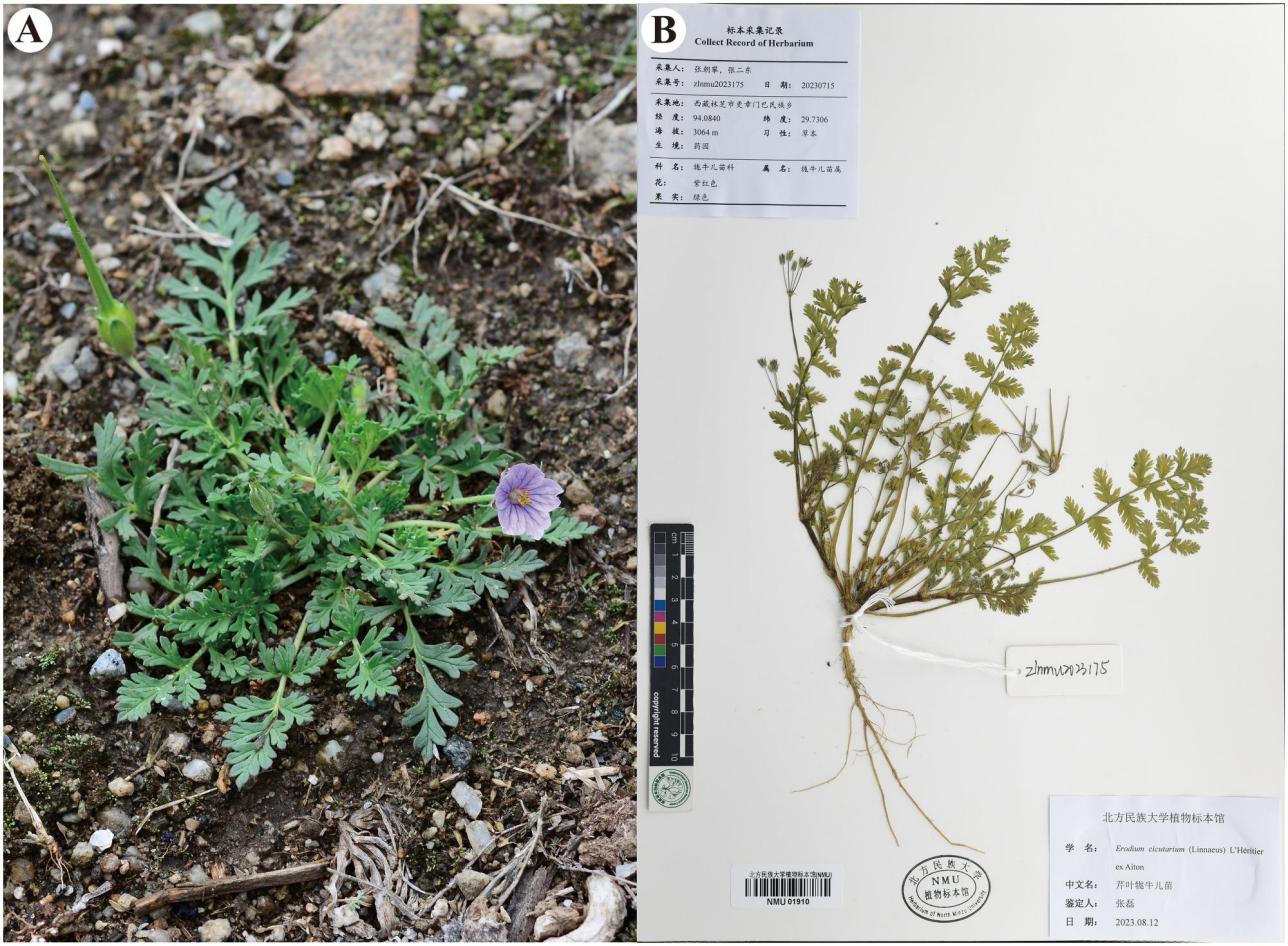
(A) An individual plant of *E. cicutarium* taken from Menba of Maizhokunggar, Linzhi, Xizang, China (photoed by Dr. Lei Zhang). (B) Herbarium of *E. cicutarium* (peduncle with 5 flowers, glandular)

Fresh leaves of *E. cicutarium* were collected from Menba in Maizhokunggar (Linzhi, Xizang, China; coordinates: 94.0840E, 29.7306 N) by Lei Zhang (zhangsanshi-0319@163.com) (Figure 1A), and subsequently desiccated using silica gel. The voucher specimen has been archived in the Herbarium of North Minzu University under the accession number of zlnmu2023175 (Figure 1B). A total of 0.5 g of the dried leaf sample was employed for DNA extraction.

## 3. Methods

Total genomic DNA was isolated with a modified CTAB method (Doyle and Doyle 1987). The NEBNext DNA Library Kit was employed to create the sequencing libraries following the manufacturer’s instructions. DNA was randomly sheared to a size of 350 bp. This library was sequenced on the Illumina NovaSeq 6000 platform with 150 bp paired-end read length. We acquired 6.2 Gb of high-quality paired-end reads for *E. cicutarium*. After eliminating the adapters, we *de Novo* assembled the cp genome *E. cicutarium* using NOVOPlasty 4.1 (Dierckxsens et al. 2017) with the specified parameters: k-mer = 39 and genome range 120,000–200,000 bp. The complete chloroplast genome sequence of *Erodium carvifolium* (HQ713469) was utilized as a reference. Plann v1.1 (Huang and Cronk 2015) was employed for annotating the chloroplast genome, and Geneious v11.0.3 (Kearse et al. 2012) was used for correcting the annotation. The sequencing depth coverage was conducted by Samtools (Li et al. 2009). To further elucidate the phylogenetic placement of *E. cicutarium* in Erodium, the chloroplast genomes of 13 representative species were retrieved from NCBI GenBank to reconstruct the chloroplast genome phylogenetic tree, with *Pelargonium alternans* as an outgroup. All the sequences were aligned using MAFFT v.7.313 (Katoh and Standley 2013). We used RAxML v8.1.24 (Stamatakis 2014) to conduct Maximum Likelihood (ML) analyses with the GTR + Γ model. The optimal model (GTR+I + G) was identified using jModeltest and Bayesian inference (BI) analysis was conducted in Mrbayes v 3.2.6 (Ronquist et al. 2012). FigTree v1.4.2 (Rambaut 2012) was subsequently utilized to visualize the phylogeny.

## 4. Results

**Figure 2.**
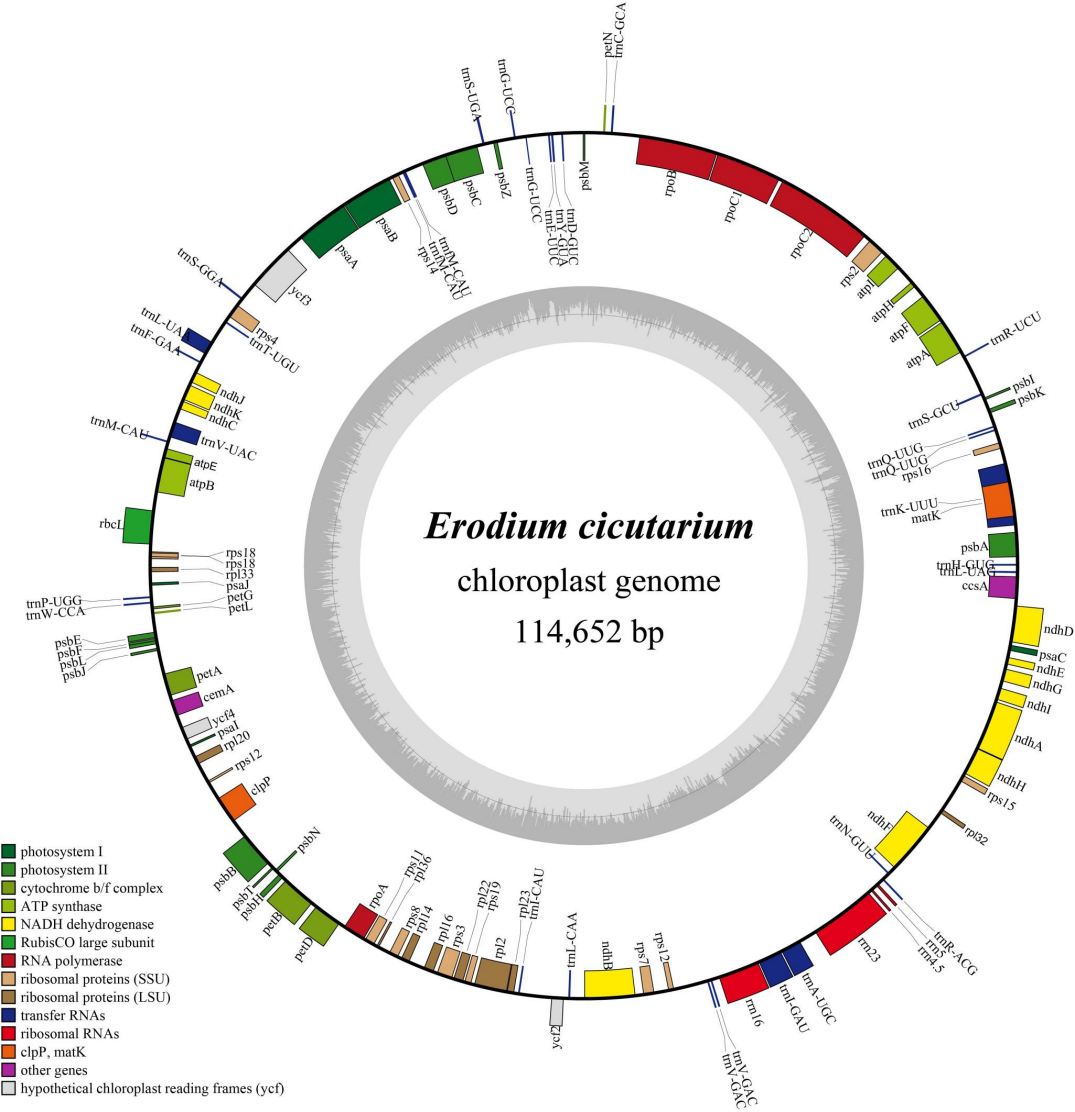
The complete chloroplast genome map of *E. cicutarium*. GC content and at content variations are colored darker gray and lighter gray is shown in the inside track, respectively. Color codes represent different functional gene groups. Genes lying inside and outside the outer circle are transcribed clockwise and counterclockwise on the outer track, respectively

**Figure 3.**
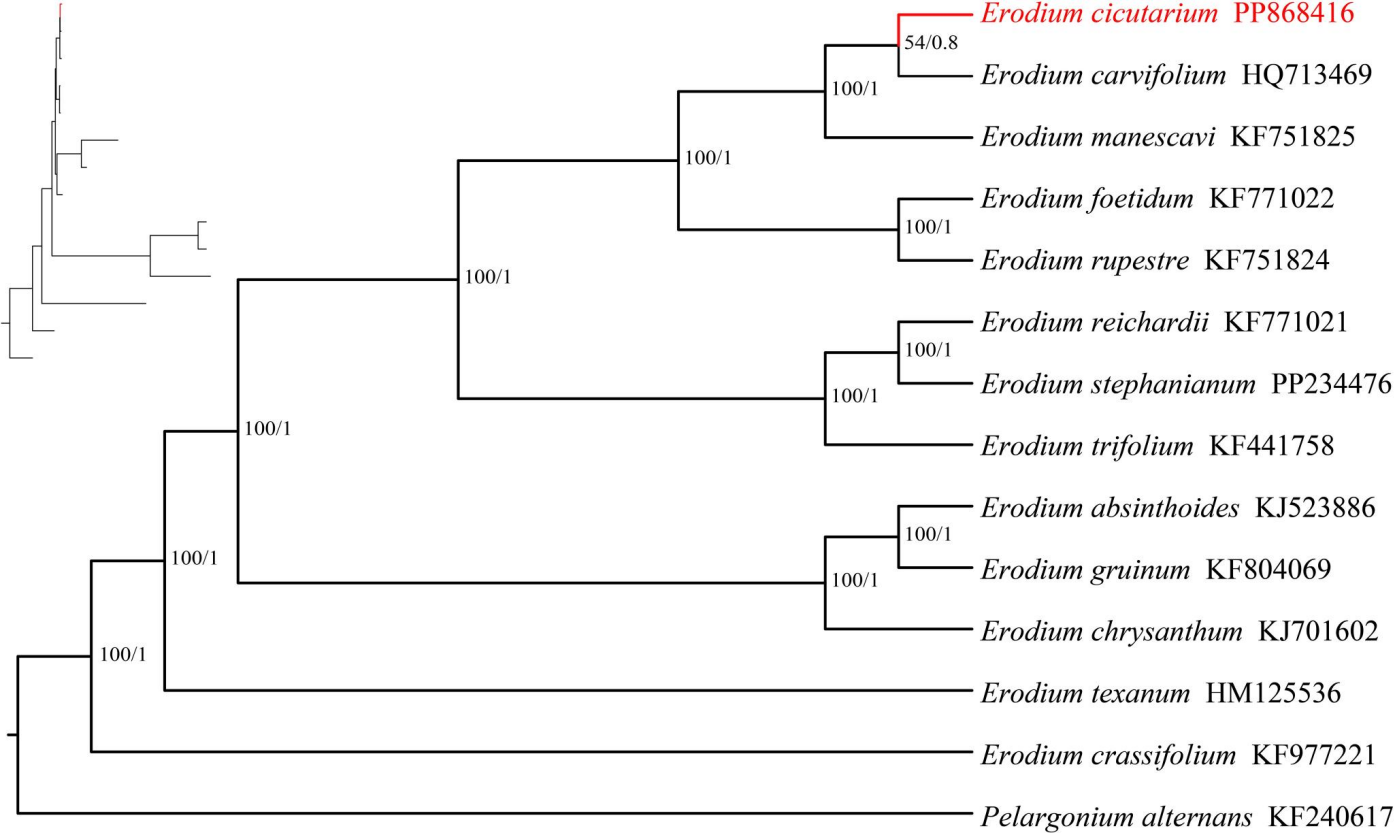
Phylogenetic tree obtained using the maximum likelihood (ML) and Bayesian inference (BI) methods of *Erodium* species based on the whole chloroplast genome. GenBank accession numbers: *Erodium absinthoides* KJ523886 (Lu et al. 2020), *Erodium carvifolium* HQ713469 (Blazier et al. 2016), *Erodium cicutarium* PP868416, *Erodium crassifolium* KF977221 (Gu et al. 2018), *Erodium foetidum* KF771022 (Zhang et al. 2016), *Erodium chrysanthum* KJ701602 (Lu et al. 2020), *Erodium gruinum* KF804069 (Blazier et al. 2016), *Erodium manescavi* KF751825 (Lee et al. 2016), *Erodium reichardii* KF771021 (Blazier et al. 2016), *Erodium rupestre* KF751824 (Blazier et al. 2016), *Erodium stephanianum* PP234476, *Erodium texanum* HM125536 (Blazier et al. 2016), *Erodium trifolium* KF441758 (Blazier et al. 2016), *Pelargonium alternans* KF240617.

After quality control and preprocessing, we obtained at least 4 gigabases (Gb) of whole-genome sequencing data. The clean reads were used to assemble high-quality chloroplast genomes through a reference-guided approach. The total chloroplast genome of *E. cicutarium* (PP868416) was 114,652 bp long, with average depths of 5884 x, 3030.51 x, and 1360 x for maximal, minimal, and average, respectively (Figure S1). It exhibits a special structural organization, consisting of only a single copy (Figure 2, Figure S2). The chloroplast genome harbored 111 complete genes (Table S1), including 75 protein-coding genes (75 PCGs), 4 ribosomal RNA genes (4 rRNAs), and 32 tRNA genes (32 tRNAs). In the genome, 12 protein-coding genes (*ndh*A, *ndh*B, *rpl*2, *pet*D, *pet*B, *clp*P, *rps*12, *rpo*C1, *atp*F) and 5 tRNA genes (*trn*A-UGC, *trn*I-GAU, *trn*K-UUU, *trn*L-UAA and *trn*V-UAC) contain an intron. Only, *ycf*3 contain two introns (Table S1). Additionally, the chloroplast genome contained 1 trans-splicing gene (Figure S3) and 9 cis-splicing genes (Figure S4). The GC content of the chloroplast DNA was 39.1%. Both ML and BI trees confirmed the placement of *E. cicutarium* within the *Erodium* family (Figure 3). In these trees, the 13 *Erodium* species are divided into three main clades with strong support (BS, PP = 100%, 1) (Figure 3). *E. cicutarium* and *E. carvifolium* formed a monophyletic groups (BS, PP =54%, 0.8), suggesting a close relationship between the two species.

## 5. Discussion and conclusion

Previous research has indicated that the CPG of seed plants ranging in size from 107 kb in Pinaceae to 170 kb in Geraniaceae with the IR region typically spanning 20–30 kb (Zhang et al. 2012; Daniell et al. 2016). In this study, we assembled the complete cp genome of *E. cicutarium,* which exhibits a total sequence length of 114,652 bp, at the smaller end of the spectrum for seed plant organelle genomes. The GC content of *E. carvifolium* is 39.1%, which is higher in Geraniaceae chloroplast genomes relative to seed plants (Wu et al. 2020). Notably, we observed an intriguing phenomenon, the *E. cicutarium* does not display a typical quadri-partite structural organization due to the absence of an IR region. This is lead to absence of genes such as ycf1 and ycf2 in the *E. cicutarium* cp genome. Loss of the IR was previously documented in two Erodium species: *E. texanum* (Guisinger et al. 2011) and *E. carvifolium* (Weng et al. 2014), indicating it is a common trait in the *Erodium* species.

The cp genome serves as a focal point in molecular biology research and has shown substantial power in solving phylogenetic relationships among angiosperms (Yang et al. 2022; Ran et al. 2024). In this study, a phylogenetic tree was developed utilizing the BI method and the ML method. The tree revealed that the cp genomes of *Erodium* species divided into three main clades with strong support. *E. cicutarium* and *E. carvifolium* forming a monophyletic group, this phylogenetic results was consistent with Fiz et al. (2006) and Alarcón et al. (2011). The alignments indicated high sequence similarity between the CPGs of *E. cicutarium* and *E. carvifolium* (Figure S5), this may explain the support value for the monophyletic group of *E. cicutarium* and *E. carvifolium* (BS, PP =54%, 0.8). Further investigation of *E. cicutarium* is necessary, including additional studies at the population level and genome analysis. Only through in-depth research can understand the structural variation of the chloroplast genome of *E. cicutarium*. In addition, expanding the collection of *Erodium* cp genomes will provide a deeper insights into the evolution of this important genus.

## Supplementary Material

Supplementary File.docx

## Data Availability

The sequenced data supporting the findings of this study are openly available in NCBI (https://www.ncbi.nlm.nih.gov/) under the accession no. PP868416. The associated BioProject, SRA and Bio-Sample numbers are PRJNA1123318, SRR29384173 and SAMN41808628, respectively.
