# Supplementary material for: The complete chloroplast genome of Erodium cicutarium (Linnaeus) l’ Héritier ex Aiton 1789 (Geraniaceae): genome characterization and phylogenetic consideration
Source: Mitochondrial DNA B Resour. 2024 Oct 24;9(10):1460–5. doi: 10.1080/23802359.2024.2420847 (PMC11504179; doi:10.1080/23802359.2024.2420847)
Supplement: Supplementary File.docx [file TMDN_A_2420847_SM2581.docx]

**Table S1** Genes present in the *Erodium carvifolium* cp genome

| Group of genes | Name of genes |
| --- | --- |
| Subunits of ATP synthase | *atp*A*, atp*B*, atp*E*, atp*F**, atp*H*, atp*I |
| Subunits of NADH-dehydrogenase | *ndh*A**, ndh*B**, ndh*C*, ndh*D*, ndh*E*, ndh*F*, ndh*G*, ndh*H*, ndh*I*, ndh*J*, ndh*K |
| Subunits of cytochrome b/f complex | *pet*A*, pet*B**, pet*D**, pet*G*, pet*L*, pet*N |
| Subunits of photosystem I | *psa*A*, psa*B*, psa*C*, psa*I*, psa*J |
| Subunits of photosystem II | *psb*A*, psb*B*, psb*C*, psb*D*, psb*E*, psb*F*, psb*H*, psb*I*, psb*J*, psb*K*, psb*L*, psb*M*, psb*N*, psb*T*, psb*Z |
| Large subunit of ribosome | *rpl*14*, rpl*16*, rp*l2**, rpl*20*, rpl*22*, rpl*23*, rpl*32*, rpl*33*, rpl*36 |
| Small subunit of ribosome | *rps*11*, rps*12**, rps*14*, rps*15*, rps*16*, rps*18*, rps*19*, rps*2*, rps*3*, rps*4*, rps*7*, rps*8 |
| DNA dependent RNA polymerase | *rpo*A*, rpo*B*, rpo*C1**, rpo*C2 |
| Subunit of rubisco | *rbc*L |
| c-type cytochrom synthesis gene | *ccs*A |
| Envelop membrane protein | *cem*A |
| Maturase | *mat*K |
| Protease | *clp*P*** |
| Conserved open reading frames | *ycf*3***,ycf*4 |
| tRNA | *trnA-*UGC**, trnC-*GCA*, trnD-*GUC*, trnE-*UUC*, trnF-*GAA*, trnfM-*CAU(x2)*, trnG-*UCC(x2)*, trnH-*GUG*, trnI-*CAU*, trnI-*GAU**, trnK-*UUU**, trnL-*CAA*, trnL-*UAA**, trnL-*UAG*, trnM-*CAU*, trnN-*GUU*, trnP-*UGG*, trnQ-*UUG(x2)*, trnR-*ACG*, trnR-*UCU*, trnS-*GCU*, trnS-*GGA*, trnS-*UGA, *trnT-*UGU*, trnV-*GAC(x2)*, trnV-*UAC**, trnW-*CCA*, trnY-*GUA |
| rRNA | *rrn*16*, rrn*23*, rrn*4.5*, rrn*5 |

Genes with one or two introns are indicated by one(*) or two asterisks (**), respectively. Two gene copies are folowed by the (X2) symbol.


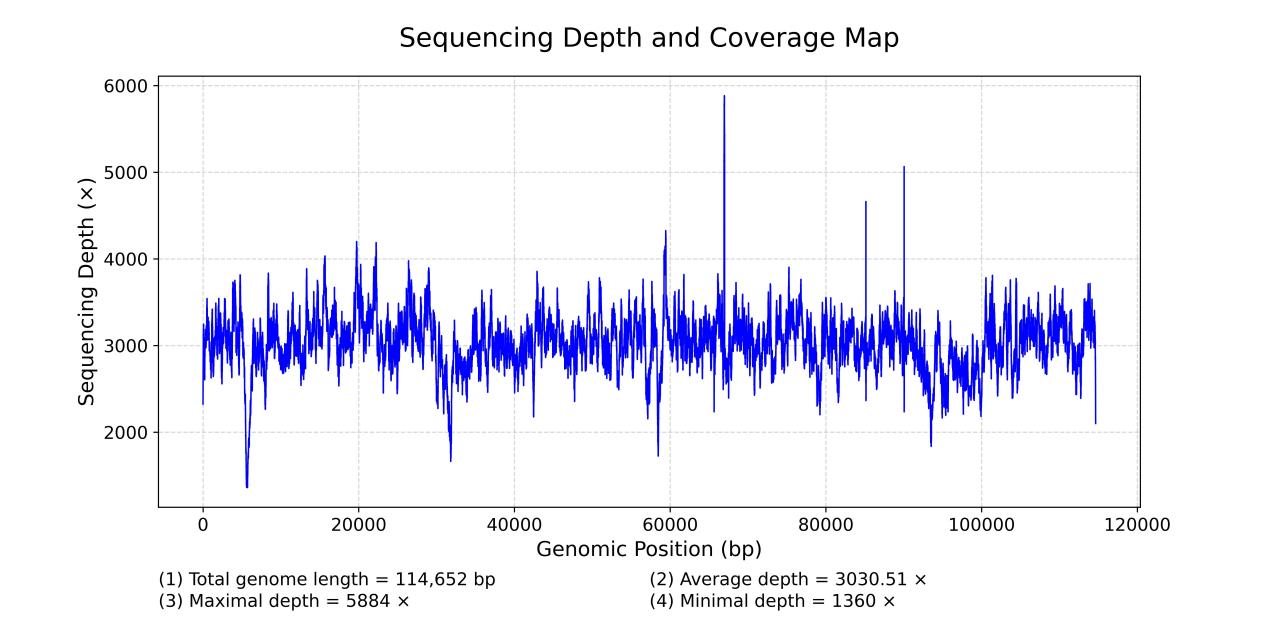


**Figure S1** Coverage depth distribution of the *Erodium cicutarium* cp genome.


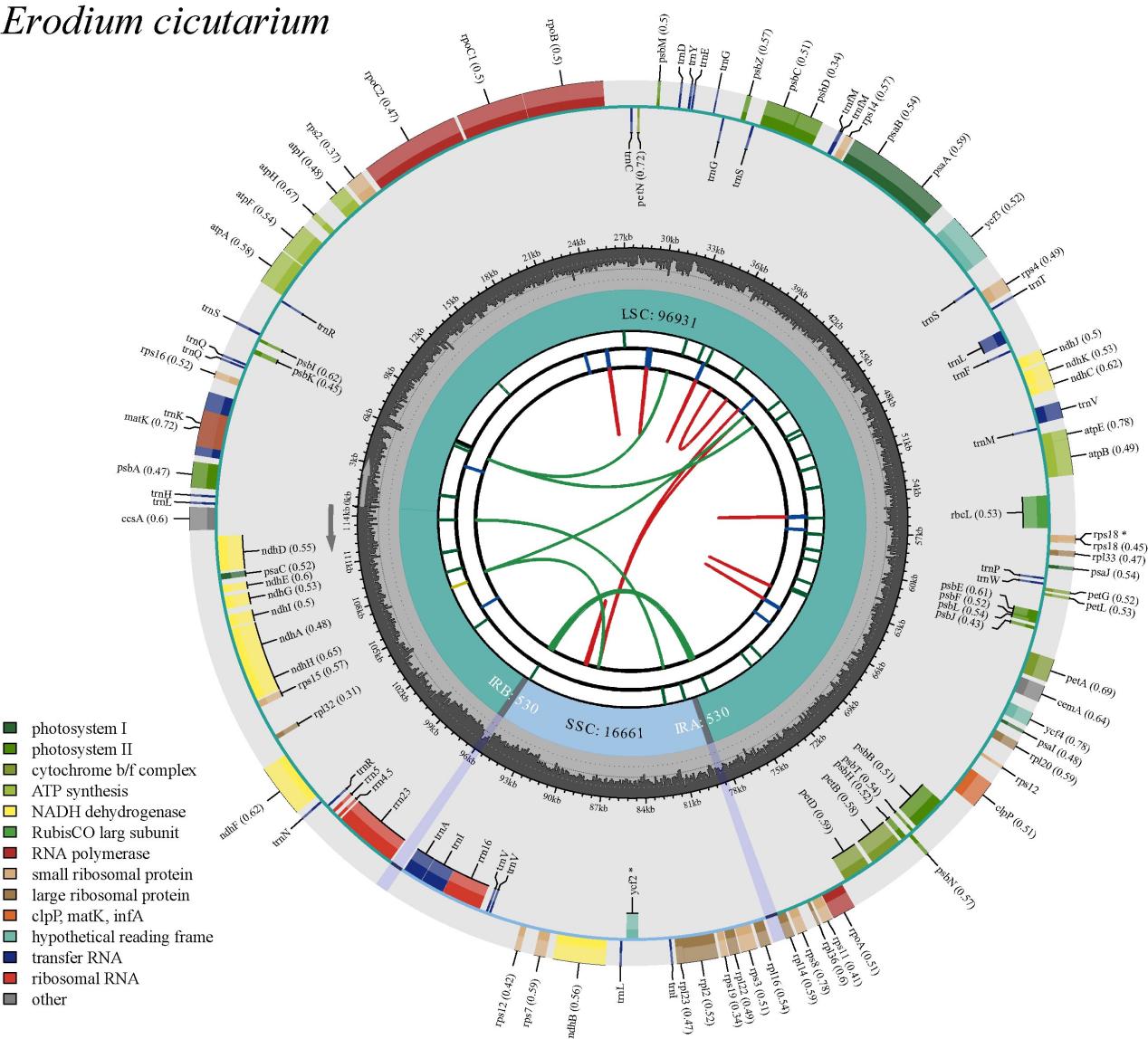


**Figure S2** Collinearity map of *Erodium cicutarium* cp genome.


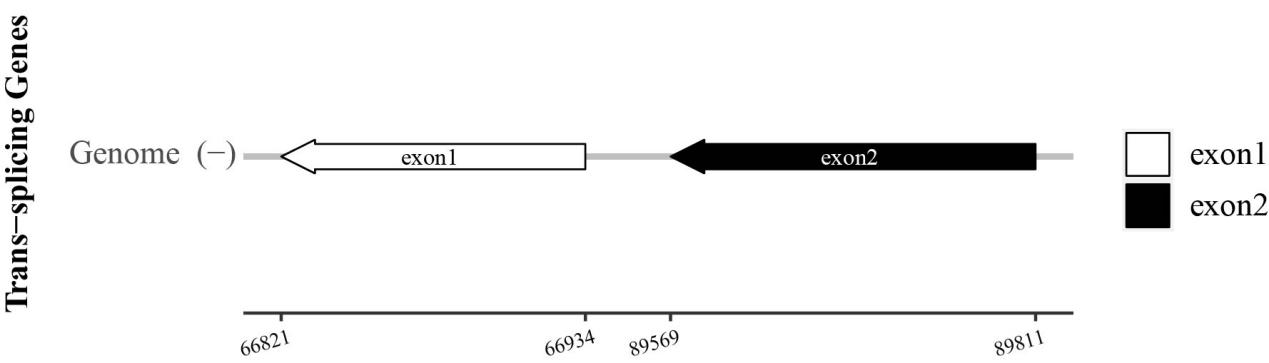


**Figure S3**. Structure of trans-splicing genes in the *Erodium cicutarium* cp genome.


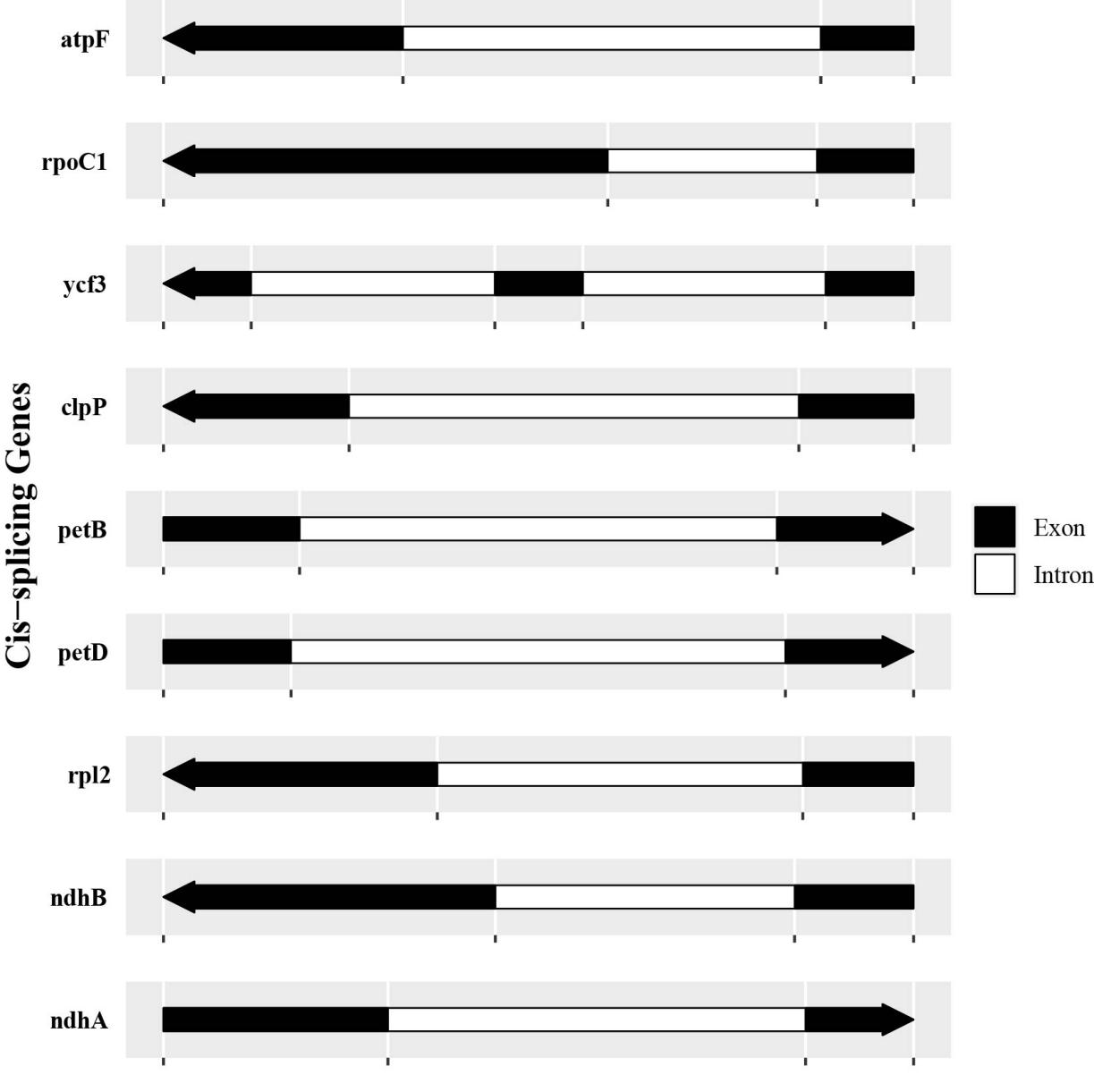


**Figure S4** Structure of Cis-splicing genes in the *Erodium cicutarium* cp genome.


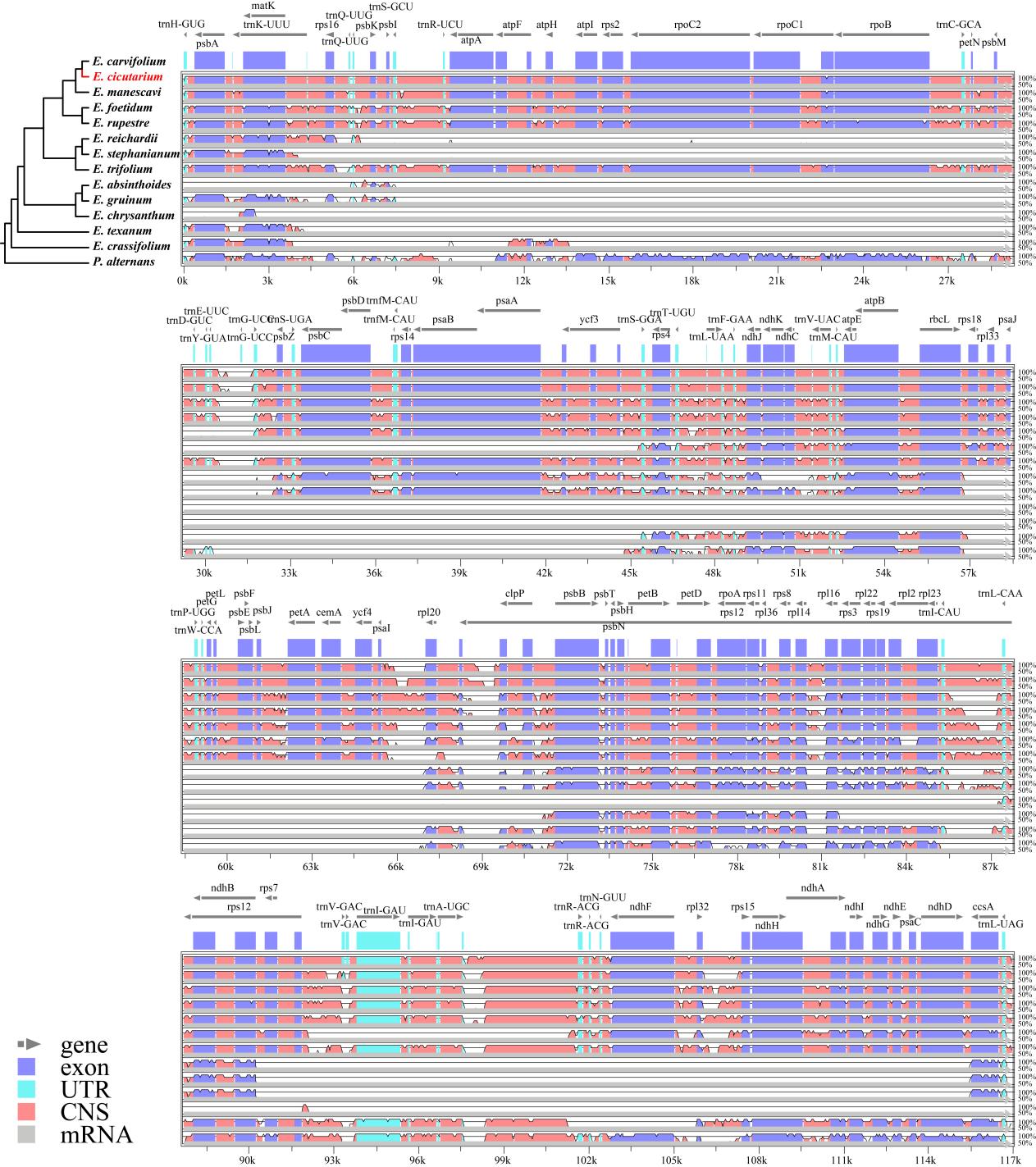


**Figure S5** Sequence alignment of the CPGs of 13 *Erodium* species. The alignment was performed using the mVISTA program and the *Pelargonium alternans* chloroplast genome was used as a reference. The y-axis indicated the degree of identity ranging from 50 to 100%. Coding and non-coding regions were marked in blue and red, respectively. Black arrows indicated the position and direction of each gene. CNS: conserved non-coding sequences.
